# The Inverse Log-Rank Test: A Versatile Procedure for Late Separating Survival Curves

**DOI:** 10.3390/ijerph20247164

**Published:** 2023-12-11

**Authors:** Jimmy T. Efird

**Affiliations:** 1 VA Cooperative Studies Program Coordinating Center, Boston, MA 02111, USA; jimmy.efird@stanfordalumni.org; 2 School of Medicine, Case Western Reserve University, Cleveland, OH 44106, USA

**Keywords:** inverse log-rank test, clinical trials, survival analysis, non-proportional hazards, delayed treatment effects

## Abstract

Often in the planning phase of a clinical trial, a researcher will need to choose between a standard versus weighted log-rank test (LRT) for investigating right-censored survival data. While a standard LRT is optimal for analyzing evenly distributed but distinct survival events (proportional hazards), an appropriately weighted LRT test may be better suited for handling non-proportional, delayed treatment effects. The “a priori” misspecification of this alternative may result in a substantial loss of power when determining the effectiveness of an experimental drug. In this paper, the standard unweighted and inverse log-rank tests (iLRTs) are compared with the multiple weight, default Max-Combo procedure for analyzing differential late survival outcomes. Unlike combination LRTs that depend on the arbitrary selection of weights, the iLRT by definition is a single weight test and does not require implicit multiplicity correction. Empirically, both weighted methods have reasonable flexibility for assessing continuous survival curve differences from the onset of a study. However, the iLRT may be preferable for accommodating delayed separating survival curves, especially when one arm finishes first. Using standard large-sample methods, the power and sample size for the iLRT are easily estimated without resorting to complex and timely simulations.

## 1. Introduction

Delayed treatment effects are the most common type of non-proportional hazards arising in clinical trials, most notably for immunologic cancer drugs [1,2,3,4]. A certain period of exposure may be necessary before achieving a treatment response, owing to the mechanism of action for compounds like PD-1 or PD-L1 inhibitors. A small insignificant difference between survival curves typically is observed initially, or, in some cases, the curves may even cross-over up to a certain time point. Thereafter, the curves diverge and late separation occurs, manifesting a differential treatment effect. While a standard log-rank test (LRT) will remain valid for rejecting the null hypothesis of no survival difference and will control the Type I error rate, the procedure will not be uniformly most powerful when the hazards for the curves are non-proportional, as is the case in late-separating curves [5]. Importantly, power may not necessarily increase as the sample size becomes larger. 

An LRT that assigns greater weight to events occurring later in the trial will be more sensitive to delayed treatment effects [6]. However, in the absence of “a priori” knowledge, finding a combination of weights that is best able to collectively accommodate various survival scenarios has been challenging [7]. Non-proportional hazards owing to differential censoring between treatment groups also poses a concern, especially when the censoring occurs with greater frequency toward the later part of the trial [8,9]. 

The inverse log-rank test (iLRT) is a computationally simple, single weight procedure that is moderately robust in detecting late occurring survival differences. Yet, this test also performs well under proportional hazards. We provide empirical examples to illustrate the novelty and versatility of this method in comparison with the multiple weight “Max-Combo” procedure and the combinatoric-based “Split-Range” test.

## 2. Materials and Methods

### 2.1. Preliminaries

#### 2.1.1. Hypergeometric Framework for Survival Time Data

Let time ($t_{i}$) ≥ 0 represent the pooled times in which participants in either Group 1 or Group 2 experience an event, respectively. Consider the layout in Table 1, where

$d_{i1}$ = # of events at ($t_{i}$) in Group 1;
$d_{i2}$ = # of events at ($t_{i}$) in Group 2;
$d_{i}\;\;\;$= ${\;\;d}_{i1}$ + $d_{i2}$;
$R_{i1}$ = # of participants available at ($t_{i}$) in Group 1;
$R_{i2}$ = # of participants available at ($t_{i}$) in Group 2;
$R_{i}\;\;\;$= ${\;R}_{i1}$ + $R_{i2}$.

Under the null hypothesis that the sets of times in the two groups are equivalent, it follows that ($d_{i1}$), conditional on the marginal total ($d_{i}$), has a hypergeometric distribution [10,11,12]. Consisting of the sum of $\left(R_{i1}\right)$ Bernoulli trials, each with a mean of $\left(\frac{d_{i}}{R_{i}}\right),$ the hypergeometric distribution is written as [13,14]

(1) PX=xi| Ri,di,Ri1=PX=xi| Ri,Ri1,di=Ri1xi Ri−Ri1di−xi Ridi=dixi Ri−diRi1−xi RiRi1,

where the random variable $\left(x_{i}\right)\;$denotes the number of events ($d_{i1}$) in Group 1 at each time point $\left(t_{i}\right)$. In many applied examples, the events of interest are deaths (d). The value for this variable is bounded below by $max\left[0,\;R_{i1}-\left(R_{i}-d_{i}\right)\right]$ and above by $min\left[d_{i},\;R_{i1}\right].$ Given equal survival times, the probability of an event occurring at ($t_{i}$) is not contingent upon the group to which a patient belongs [15]. 

Observing that$\;\left(x_{i}\right)$ is less than or equal to $\left(R_{i1}\right),\;$the number at risk in Group 1 at $\left(t_{i}\right),$ it follows that [16]

(2) $$\;\;P\left(X=x_{i}|\;{R_{i},R_{i1},d}_{i}\right)=\frac{\prod_{j=0}^{x_{i}-1}\left(d_{i}-j\right)\prod_{j=0}^{R_{i1}-x_{i}-1}\left(R_{i}-d_{i}-j\right)}{\prod_{j=0}^{R_{i1}-1}\left(R_{i}-j\right)}.$$

#### 2.1.2. Expectation and Variance

The properties of the hypergeometric distribution are well described in the literature [13,14,17,18]. Briefly, the first raw moment for (X) gives the expected number of patients who experience an event at time ($t_{i}$) within a particular group, and is written as

(3) $$\mu_{1}^{\prime }=\mu_{x_{i}}=E\left[{X=x}_{i}\right]=\frac{R_{i1}d_{i}}{R_{i}}.$$

The finite second central moment is obtained as

(4) μ2′=Exi(xi−1)+Exi=∑xi=2Ri1didi−1di−2xi−2Ri−diRi1−xiRiRi−1Ri1Ri1−1Ri−2Ri1−2+Ri1diRi.

Subtracting the square of the first raw moment from the second central moment gives the variance of (X) at time ($t_{i}$), i.e.,

(5) $$\sigma_{x_{i}}^{2}=Var\left[{X=x}_{i}\right]=\frac{d_{i}R_{i1}R_{i2}\left(R_{i}-d_{i}\right)}{{R_{i}}^{2}\left(R_{i}-1\right)}=R_{i1}\left[\frac{d_{i}}{R_{i}}\right]\left[1-\left(\frac{d_{i}}{R_{i}}\right)\right]\left[\frac{R_{i}-R_{i1}}{R_{i}-1}\right].$$

#### 2.1.3. Large Sample Properties

Under large sampling conditions, the null distribution for the hypergeometric test may be indirectly approximated by a Gaussian distribution [19]. As ($d_{i}$) and ($R_{i}$) approach infinity (with a fixed ratio), and assuming that $\left(R_{i}-d_{i}\right)\;$is relatively large, with a fixed finite $\left(x_{i}\right)$, we see that [18]

(6) $$P\left(X=x_{i}|\;{R_{i},R_{i1},d}_{i}\right)\sim \left(\frac{d_{i}!}{\left(d_{i}-x_{i}\right)!}\right)\left(\frac{R_{i1}!}{x_{i}!\left(R_{i1}-x_{i}\right)!}\right)\left(\frac{\left(R_{i}-d_{i}\right)!}{\left(R_{i}-d_{i}-R_{i1}+x_{i}\right)!}\right)\left(\frac{\left(R_{i}-R_{i1}\right)!}{\left(R_{i}\right)!}\right)$$

(7) $$\sim \left[\frac{\Gamma \left(R_{i1}+1\right)}{\Gamma \left(x_{i}+1\right)\Gamma \left(R_{i1}-x_{i}+1\right)}\right]\left[{\left(\frac{d_{i}}{R_{i}}\right)}^{x_{i}}\left\{\frac{{\left(R_{i}-d_{i}\right)}^{R_{i1}-x_{i}}}{{\left(R_{i}\right)}^{R_{i1}-x_{i}}}\right\}\right],$$

(8) $$\;\sim \left[\left\{\frac{\prod_{j=1}^{x_{i}}\left(R_{i1}-j+2\right)}{\prod_{j=0}^{x_{i}-1}\left(x_{i}-j\right)}\right\}-\left\{\frac{\prod_{j=1}^{x_{i}-1}\left(R_{i1}-j+1\right)}{\prod_{j=0}^{x_{i}-2}\left(x_{i}-1-j\right)}\right\}\right]\left[{\left(\frac{d_{i}}{R_{i}}\right)}^{x_{i}}\left\{{\left(\frac{R_{i}-d_{i}}{R_{i}}\right)}^{R_{i1}-x_{i}}\right\}\right],\;$$

where the term in the left square brackets of the last two expressions denotes the unordered ways to choose $\left(x_{i}\right)$ from a set of $\left(R_{i1}\right)$ elements (Pascal’s pyramid) [20]. The approximation becomes increasingly better as the ratio terms Ri12Ri, xi2di, and Ri1−xi2Ri−di diminish in size. Applying Stirling’s approximation,

(9) $$\gamma !\sim {\left(\frac{\gamma }{e}\right)}^{\gamma }\sqrt{2\pi \gamma }\;\;\;\;\left(with\;a\;positive\;relative\;error\leq \frac{1}{12\gamma -1}\right),$$

we have [21]

(10) $$\;P\left(X=x_{i}|\;{R_{i},R_{i1},d}_{i}\right)\;\sim \left[\frac{{\left(\frac{R_{i1}}{e}\right)}^{R_{i1}}\sqrt{2\pi R_{i1}}}{{\left(\frac{x_{i}}{e}\right)}^{x_{i}}\sqrt{2\pi x_{i}}{\left(\frac{R_{i1}-x_{i}}{e}\right)}^{R_{i1}-x_{i}}\sqrt{2\pi \left(R_{i1}-x_{i}\right)}}\right]{\left(\frac{d_{i}}{R_{i}}\right)}^{x_{i}}{\left(1-\frac{d_{i}}{R_{i}}\right)}^{R_{i1}-x_{i}}$$

(11) $$\sim \;{\left[\frac{R_{i1}d_{i}}{R_{i}x_{i}}\right]}^{x_{i}}{\left[\frac{R_{i1}\left({R_{i}-d}_{i}\right)}{R_{i}\left({R_{i1}-x}_{i}\right)}\right]}^{R_{i1}-x_{i}}\sqrt{\frac{R_{i1}}{2\pi x_{i}\left({R_{i1}-x}_{i}\right)}}.$$

Next, we obtain the following identities

(12) $$-log\left(1+\frac{R_{i}x_{i}-R_{i1}d_{i}}{R_{i1}d_{i}}\right)=-log\left(\frac{x_{i}}{R_{i1}\left(\frac{d_{i}}{R_{i}}\right)}\right)=log\left(\frac{R_{i1}d_{i}}{R_{i}x_{i}}\right)$$

(13) $$\;\Rightarrow log\left(\frac{R_{i1}\left({R_{i}-d}_{i}\right)}{R_{i}\left({R_{i1}-x}_{i}\right)}\right)=-log\left(1-\frac{x_{i}-R_{i1}\left(\frac{d_{i}}{R_{i}}\right)}{R_{i1}\left(1-\frac{d_{i}}{R_{i}}\right)}\right)=-log\left(1-\frac{R_{i}x_{i}-R_{i1}d_{i}}{R_{i1}\left({R_{i}-d}_{i}\right)}\right).$$

Noting that

(14) $$-\sum_{j=1}^{\infty }\frac{{\left(-1\right)}^{j}a^{-j}}{j}=log\left(1+a\right)\;\mathrm{for}\;|a|>1$$

and combining terms, with $O\left(\frac{{\left(x_{i}-R_{i1}\left(d_{i}/R_{i}\right)\right)}^{3}}{{R_{i1}}^{2}}\right)\;$dominating over $O\left(\frac{{\left(x_{i}-R_{i1}\left(d_{i}/R_{i}\right)\right)}^{3}}{{R_{i1}}^{3}}\right),$ it follows that

(15) $${\left[\frac{R_{i1}d_{i}}{R_{i}x_{i}}\right]}^{x_{i}}{\left[\frac{R_{i1}\left({R_{i}-d}_{i}\right)}{R_{i}\left({R_{i1}-x}_{i}\right)}\right]}^{R_{i1}-x_{i}}=exp\left(-\frac{1}{2}{\left[\frac{x_{i}-R_{i1}\left(\frac{d_{i}}{R_{i}}\right)}{\sqrt{R_{i1}\left(\frac{d_{i}}{R_{i}}\right)\left(1-\frac{d_{i}}{R_{i}}\right)}}\right]}^{2}\right).$$

Continuing, we assume that

(16) $$x_{i}-R_{i1}\left(\frac{d_{i}}{R_{i}}\right)\approx \sqrt{R_{i1}}.$$

When $\left(\frac{d_{i}}{R_{i}}\right)\;$is neither close to 0 nor 1 and both $\left[R_{i1}\left(\frac{d_{i}}{R_{i}}\right)\right]\;$and $\left[R_{i1}\left(1-\left(\frac{d_{i}}{R_{i}}\right)\right)\right]$ are large, the application of L’Hôpital’s rule shows that

(17) $$\underset{R_{i1}\to \infty }{\lim}\left[\frac{R_{i1}-R_{i1}\left(\frac{d_{i}}{R_{i}}\right)}{x_{i}}\right]=\underset{R_{i1}\to \infty }{\lim}\left[\frac{1-\left(\frac{d_{i}}{R_{i}}\right)}{\frac{1}{2\sqrt{R_{i1}}}+\left(\frac{d_{i}}{R_{i}}\right)}\right]\to 1.$$

Therefore,

(18) $$R_{i1}-x_{i}\approx R_{i1}\left(1-\left(\frac{d_{i}}{R_{i}}\right)\right)-\sqrt{R_{i1}}\left[\frac{R_{i1}\left(1-\left(\frac{d_{i}}{R_{i}}\right)\right)}{x_{i}}\right]$$

(19) $$\;⟹x_{i}\left(R_{i1}-x_{i}\right)\approx \left(x_{i}-\sqrt{R_{i1}}\right)\left[R_{i1}\left(1-\left(\frac{d_{i}}{R_{i}}\right)\right)\right]\approx {\left(R_{i1}\right)}^{2}\left(\frac{d_{i}}{R_{i}}\right)\left(1-\left(\frac{d_{i}}{R_{i}}\right)\right).$$

Substituting accordingly and combining terms gives

(20) $$P\left(X=x_{i}|\;{R_{i},R_{i1},d}_{i}\right)\;\sim \sqrt{\frac{1}{2\pi R_{i1}\left(\frac{d_{i}}{R_{i}}\right)\left(1-\left(\frac{d_{i}}{R_{i}}\right)\right)}}exp\left(-\frac{1}{2}{\left[\frac{x_{i}-R_{i1}\left(\frac{d_{i}}{R_{i}}\right)}{\sqrt{R_{i1}\left(\frac{d_{i}}{R_{i}}\right)\left(1-\frac{d_{i}}{R_{i}}\right)}}\right]}^{2}\right)\;\sim \;N\left(\mu_{x_{i}},\sigma_{x_{i}}^{2}\right),$$

where $\mu_{x_{i}}$=$\left(\frac{{R_{i1}d}_{i}}{R_{i}}\right)$ and $\sigma_{x_{i}}^{2}=R_{i1}\left(\frac{d_{i}}{R_{i}}\right)\left(1-\frac{d_{i}}{R_{i}}\right).$ Therefore, the discrete probability elements for each (X) at time ($t_{i}$) shrink infinitesimally to yield a symmetrical continuous density centered at $\left(\mu_{x_{i}}\right)$ with asymptotic points of inflection at $\left(\mu_{x_{i}}\pm \;\sigma_{x_{i}}^{2}\right).$ A simple transformation gives

(21) $$\xi_{i}=\frac{\left(x_{i}-\mu_{x_{i}}\right)}{\sigma_{x_{i}}}\;\sim \;N(0,\;1).$$

Noting that limRi→∞⁡Ri13Ri2→0, the $\left[\frac{R_{i}-R_{i1}}{R_{i}-1}\right]$ term in variance for the hypergeometric distribution asymptotically approaches unity, and, as expected, the corresponding variance for the Gaussian distribution becomes $\left[R_{i1}\left[\frac{d_{i}}{R_{i}}\right]\left[1-\left(\frac{d_{i}}{R_{i}}\right)\right]\right]$. Lastly, we mention that a more direct proof yielding the normal distribution can be obtained by rewriting the binomial coefficients in the hypergeometric distribution using de-Moivre Laplace’s asymptotic formula and simplifying [22].

#### 2.1.4. Useful Approximations, Bounds, and Recursive Formulas

When $\left(R_{i}>50\right),\left(d_{i}\leq R_{i1}\right),and\left(\frac{{2d}_{i}-\eta }{{{2R}_{i}-R}_{i1}+1}\leq 1\right),\;$a reasonable approximation for the sum of hypergeometric terms, in terms of the Bernoulli distribution, is given as [14]

(22) PX≤r| Ri,di,Ri1=∑xi=max0, Ri1−Ri−dirdixi Ri−diRi1−xi RiRi1

(23) $$=1-\sum_{x_{i}=r+1}^{R_{i1}}\left[\left[\frac{\prod_{j=1}^{x_{i}}\left(R_{i1}-j+2\right)}{\prod_{j=0}^{x_{i}-1}\left(x_{i}-j\right)}\right]-\left[\frac{\prod_{j=1}^{x_{i}-1}\left(R_{i1}-j+1\right)}{\prod_{j=0}^{x_{i}-2}\left(x_{i}-1-j\right)}\right]\right]\left[{\left(\frac{d_{i}}{R_{i}}\right)}^{x_{i}}\left[\frac{{\left(R_{i}-d_{i}\right)}^{R_{i1}-x_{i}}}{{\left(R_{i}\right)}^{R_{i1}-x_{i}}}\right]\right]\;$$

(24) $$\sim 1-\sum_{x_{i}=r+1}^{R_{i1}}\left[\left[\frac{\prod_{j=1}^{x_{i}}\left(R_{i1}-j+2\right)}{\prod_{j=0}^{x_{i}-1}\left(x_{i}-j\right)}\right]\left[\frac{\prod_{j=1}^{x_{i}-1}\left(R_{i1}-j+1\right)}{\prod_{j=0}^{x_{i}-2}\left(x_{i}-1-j\right)}\right]\right]\left[{\left(\frac{{2d}_{i}-r}{{{2R}_{i}-R}_{i1}+1}\right)}^{x_{i}}{\left(\frac{2\left(R_{i}-d_{i}\right)+\left(1+r\right)-R_{i1}}{2R_{i}-R_{i1}+1}\right)}^{R_{i1}-x_{i}}\right].$$

A lower and upper bound for the hypergeometric density, as a function of the Bernoulli distribution is written as [18]

(25) exp−12 xixi−12di−xi−12Ri1−xiRi1−xi−1Ri−di−Ri1+xiRi1xiRi1diRixiRi−Ri1diRiRi1−xi≤dixi Ri−diRi1−xi RiRi1≤expRi1Ri1−12Ri−Ri1Ri1xiRi1diRixiRi−Ri1diRiRi1−xi.

This readily follows from the inequality

(26) exp−Ri1Ri1−12Ri−Ri1≤RiRi1RiRi1≤Ri−Ri1diRiRi1∑xi=0Ri1Ri1xiRi1diRi−Ri1dixi.

In many cases, determining hypergeometric probabilities can be challenging. A convenient recursive equation is easily derived as

(27) $$P\left(X=x_{i}+1|\;{R_{i},R_{i1},d}_{i}\right)=P\left(X=x_{i}|\;{R_{i},R_{i1},d}_{i}\right)\left[\frac{d_{i}!}{\left(x_{i}+1\right)!\left(d_{i}-x_{i}-1\right)!}\right]\left[\frac{x_{i}!\left(\left(d_{i}-x_{i}\right)!\right)}{d_{i}!}\right]\;\times \left[\frac{\left(R_{i}-d_{i}\right)!}{\left(R_{i1}-x_{i}-1\right)!\left(R_{i}-{d_{i}-R_{i1}+x}_{i}+1\right)!}\right]\left[\frac{\left(R_{i1}-x_{i}\right)!\left(R_{i}-{d_{i}-R_{i1}+x}_{i}\right)!}{\left(R_{i}-d_{i}\right)!}\right]$$

(28) $$P\left(X=x_{i}|\;{R_{i},R_{i1},d}_{i}\right)\left[\frac{\left(R_{i1}-x_{i}\right)\left(d_{i}-x_{i}\right)}{\left(x_{i}+1\right)\left(R_{i}-{d_{i}-R_{i1}+x}_{i}+1\right)}\right].$$

Rearranging, we see that

(29) $$P\left(X=x_{i}-1|{{\;R}_{i},R_{i1},d}_{i}\right)\;=P\left(X=x_{i}|\;{R_{i},d}_{i},R_{i1}\right)\left(\frac{x_{i}}{d_{i}-x_{i}+1}\right)\left(\frac{R_{i}-d_{i}-R_{i1}+x_{i}}{R_{i1}-x_{i}+1}\right).$$

### 2.2. Weighted Log-Rank Test 

Consider (m) separate event time points ($t_{1}<t_{2}<t_{i}<\cdots <t_{m}$) and let ($w_{i}$) denote a non-disjoint, positive weight function that is appropriately bounded (detectable, non-zero measure) for each $\left(i\right)$ value. The linear combination ($\sum_{i}^{m}w_{i}\xi_{i}$) yields the weighted LRT, which defaults to the standard LRT when the weight function is equal to unity for each time point [23,24]. Because the moment generating function (MGF) for this linear combination is equal to the MGF of a normal distribution with mean = ($\sum_{i}^{m}w_{i}\mu_{x_{i}}$) and variance = ($\sum_{i}^{m}{w_{i}}^{2}\sigma_{x_{i}}^{2}$), i.e.,

(30) $${MGF}_{X}\left(s\right)=e^{\left[s\left(\sum_{i=1}^{m}w_{i}\mu_{x_{i}}\right)+\frac{s^{2}}{2}\left(\sum_{i=1}^{m}{w_{i}}^{2}\sigma_{x_{i}}^{2}\right)\right]},$$

it holds that

(31) $$\sum_{i=1}^{m}w_{i}\xi_{i}\sim N\;(\sum_{i=1}^{m}w_{i}\mu_{x_{i}},\;\sum_{i=1}^{m}{w_{i}}^{2}\sigma_{x_{i}}^{2}),$$

since no distinct probability distributions can have the same moment generating function. Thus, under large sampling conditions, the summation of ($\;w_{i}\xi_{i}$) over $\left(m\right)$ time points has an approximate standard normal distribution, i.e., N(0, 1) or, equivalently, by taking the square, a chi-square distribution with one degree of freedom. 

Rewriting the weighted LRT as

(32) $$\xi_{w_{i}}=\sum_{i=1}^{m}\frac{{\left[\left(w_{i}\right)\left(d_{i1}-\frac{R_{i1}d_{i}}{R_{i}}\right)\right]}^{2}\;}{\sum_{i=1}^{m}{w_{i}}^{2}\sigma_{x_{i}}^{2}}=\sum_{i=1}^{m}\frac{{\left[w_{i}\left(\;O_{i}-E_{i}\right)\right]}^{2}}{\sum_{i=1}^{m}Var\left[w_{i}\left(O_{i}-E_{i}\right)\right]},$$

where $\left(O_{i}-E_{i}\right)$ denotes the deviation of the observed values $\left(d_{i1}\right)$ from their expected values, we see that the numerator of ($\xi_{w_{i}}$) corresponds to the weighted sum of conditionally independent and uncorrelated hypergeometric (asymptotically normal) random variables, with each term having a mean of zero, under the null hypothesis of no treatment effect (i.e., $E\left[{{w_{i}d}_{i1}}_{i}\right]=\frac{{w_{i}R}_{i1}d_{i}}{R_{i}}$) [10]. Since the event times are conditionally independent of one another and are functionally predictable (i.e., $\xi_{w_{i}}$ is not contingent on outcomes that occur at or beyond $t_{i}$) [25], the variance of the numerator is simply equal to the sum of the variances for the individual $\left[w_{i}\left(O_{i}-E_{i}\right)\right]$ terms [15]. Specifically,

(33) $$\;\;\;\;\;\;\;\;\;\;\;\;\;\;\;\;\;\;\;\;Var\left[w_{i}\left(O_{i}-E_{i}\right)\right]={w_{i}}^{2}\left[Var\left(O_{i}\right)+Var\left(E_{i}\right)-2Cov(O_{i},\;E_{i})\right]={w_{i}}^{2}Var\left(O_{i}\right),$$

as both the variance of $\left(E_{i}\right)$ and the $Cov(O_{i},\;E_{i})$ are equal to zero. Of further note, $\left(\xi_{w_{i}}\right)$ remains the same if $\left(w_{i}\right)$ is multiplied or divided by a scaler constant [26,27].

Applying the conditional central limit theorem (assuming the exchangeability of elements and Lundeberg’s sufficiency conditions for martingales—finite variance, tightness, and uniform integrability), it follows that $\left(\xi_{w_{i}}\right)\;$is asymptotically consistent and weakly convergent in distribution to a chi-square distribution with 1 degree of freedom, even when the individual terms are not necessarily identically distributed [28,29,30,31,32,33]. Thus, the conditional central limit theorem aligns with the abovementioned MGF approach for defining the large sample distribution of $\left(\xi_{w_{i}}\right)\;$but with less stringent conditions that are better suited for real-world applications [34]. Nonetheless, the small-sample behavior in both scenarios may be difficult to anticipate in practice, especially for highly censored and sparse tailed data [35].

### 2.3. Selection of Weights

Various choices for ($w_{i}$) have been proposed in the literature. A popular selection is to set ($w_{i}$) equal to 1, which gives the standard Mantel–Haenszel LRT without continuity correction [23]. While this option is fairly robust for detecting survival curve differences, especially in the case of proportional hazards, there is no universal consensus regarding the best weight or combination of weights to use when the hazards (for the two groups under comparison) are not constant over time, as is the case for late separating survival curves. One flexible option is the two-parameter Fleming–Harrington (FH) weight, with ($w_{i}$) defined as

(34) $$\;G(\rho ,\;\gamma )=\left\{{\tilde{S}\left(t-\right)}^{\rho }\right\}\left\{1-{\tilde{S}\left(t-\right)}^{\gamma }\right\},\;$$

where $\tilde{S}\left(t-\right)$ is the left-continuous product-limit estimate, and $\left(\rho \geq 0,\;\lambda \geq 0\right)$ [29]. Here, G(ρ=0,γ=0), G(ρ>0,γ=0), G(ρ>0,γ>0), and G(ρ=0,γ>0) purportedly corresponds to “evenly distributed”, “early”, “mid”, and “late” treatment effects, with G(ρ=0,γ=0) denoting the standard LRT and G(ρ=1,γ=0) denoting the Prentice–Wilcoxon statistic. Barring prior knowledge, the selection of (ρ) and (γ) is largely arbitrary. Arguably, certain weights may lack clinical relevance, focusing only on a specific portion of a survival curve with low event rates or diminishing treatment effects. 

A compromise entails taking the maximum of the standardized statistics for a preset combination of FH-LRT values for (ρ) and (γ). Dividing the difference vector by the corresponding square root of Fisher’s information matrix (a non-singular, uniformly minimum variance unbiased estimator), the resultant statistic asymptotically assumes a multivariate Gaussian distribution [36]. Known as the “Max-Combo” method, the test accommodates various treatment effects by selectively up- or down-weighting the log-rank statistics over time [37]. In general, combination approaches are more powerful than the standard LRT under a range of nonproportional hazard conditions [38,39]. The critical value $\left(c_{\alpha }\right)\;$for a $\left(k\right)$-component Max-Combo test ($Z_{Max}^{k})$ is defined such that

(35) $$P\left[max\left\{Z_{1,\;\;}Z_{1,\;\;}\ldots \;Z_{k,\;\;}\right\}\geq c_{\alpha }\right]=\alpha .$$

Commonly used combinations include

(36) $$Z_{Max}^{3}=max\left\{G\left(0,0\right),G\left(1,0\right),G\left(0,1\right)\right\};\;$$

(37) $$Z_{Max}^{4}=max\left\{G\left(0,0\right),G\left(0,1\right),G\left(1,1\right),G\left(1,0\right)\right\};\;$$

(38) $$Z_{Max}^{4}=max\left\{G\left(0,0\right),G\left(2,0\right),G\left(0,2\right),G(2,2)\right\},$$

with the first abovementioned $Z_{Max}^{4}$ traditionally being designated as the default set of weights. The Max-Combo test has been shown to perform well in many applied examples with non-proportional hazards [40]. However, under moderate to heavy censoring and noting the potentially high correlation among weighted LRTs, the family of combination procedures (including the Max-combo test) may not be more versatile than individual component LRT tests [8]. The extension to a group sequential analysis allows the Max-Combo procedure to accommodate multiple time point decisions, with the test statistic assuming a joint normal distribution under the null hypothesis (per the application of Slutsky’s theorem) [41,42,43,44].

### 2.4. Inverse Log-Rank Test 

A key constraint of the Max-Combo test in practical applications is that the null hypothesis can be rejected in favor of both the experimental and reference arms for an identical set of observations [45]. That is, when survival curves cross and one wishes to test the superiority of Treatment A, it is possible for the Max-Combo method to reject the null hypothesis in favor of Treatment A; while in contrast, if the objective is to test the superiority of Treatment B, then the Max-Combo method could conceivably yield the opposite conclusion given the same data (i.e., reject the null hypothesis in favor of Treatment B). Alternatively, the iLRT presents a single-weight LRT for analyzing non-proportional hazard survival curves [46]. 

Based on a smoothed, non-negative function of sample values that converges in probability to its true state, the inversely weighted logarithm of the combined number of patients at risk at each of (m) study time points is given by

(39) $$w_{i}=\frac{\log(R_{i})}{R_{i}}\;\;\;(i=1\;to\;m).$$

The iLRT is defined as

(40) $$\zeta_{w_{i}}=\sum_{i=1}^{m}\frac{{\left[\left(w_{i}\right)\left(d_{i1}-\frac{R_{i1}d_{i}}{R_{i}}\right)\right]}^{2}}{\sum_{i=1}^{m}{\left(w_{i}\right)}^{2}\sigma_{x_{i}}^{2}}=\sum_{i=1}^{m}\frac{{\left[\left(\frac{log(R_{i})}{R_{i}}\right)\left(d_{i1}-\frac{R_{i1}d_{i}}{R_{i}}\right)\right]}^{2}}{\sum_{i=1}^{m}{\left(\frac{log(R_{i})}{R_{i}}\right)}^{2}\sigma_{x_{i}}^{2}},\;$$

with the *p*-value (two-sided test) estimated as

(41) $$P\left(X_{1}^{2}>\zeta_{w_{i}}\right)=\int_{x=\zeta_{w_{i}}}^{\infty }\frac{e^{-x/2}}{\sqrt{2\pi x}}dx.$$

As (π) in the denominator of the last equation is equal to $\Gamma \left(1/2\right)$, the integrand corresponds to the probability density function of a chi-square distribution with 1 degree of freedom. Being a score that is statistic, which can be alternatively expressed as a discrete-time, partial likelihood function, $\left(\zeta_{w_{i}}\right)$ easily accommodates censored data [47,48]. 

### 2.5. Split-Range Test 

Consider the special case of a 2-arm, randomized clinical trial where all of the patients in the comparison arm (Group 2) achieve the event of interest by a certain time, while some of the patients in the test arm (Group 1) have survival times beyond this time point. A *p*-value for testing the null hypothesis $\left(H_{0}\right)$ of no survival differences between the groups may be computed using the split-range test (SRT) [49]. In this non-parametric method, designate the number of patients in Group 1 as $\left(n_{1}\right)$ and the number in Group 2 as $\left(n_{2}=N-n_{1}\right)$, with $\left(N\right)\;$denoting the total sample size. This is equivalent to the Fermi–Dirac “ball and cell” model, where $\left(n_{2}\right)$ balls are randomly dropped into $\left(N\right)$ cells (corresponding to ranked survival times), allowing one ball per cell. Numbering the cells from 1 to $\left(N\right)$, the range $\left(R\right)$ is defined as the number of the highest occupied cell minus the lowest occupied cell. The value for the range must be a number from $\left(n_{2}-1\right)$ to $\left(N-1\right).$ To test $\left(H_{0}\right)$ with a Type I error rate of $\left(\alpha \right)\;$for falsely rejecting the null hypothesis, find the integer $\left(\varphi \right)$ satisfying

(42) $$\sum_{r=n_{2}-1}^{\varphi }P\left(R=r\right)=\alpha$$

and reject $\left(H_{0}\right)$ if the observed value of $\left(R\right)$ does not exceed $\left(\alpha \right).$ When censored values occur in Group 1 before the last event occurs in the Group 2, then $\left(\alpha \right)$ denotes an upper bound. That is, some of the true survival times for these censored values may be longer than all the elements in Group 1. By decreasing the range, this results in a smaller *p*-value. 

Analogously, the split-range test can be applied in reverse by randomly dropping the $\left(n_{1}\right)$ balls into the $\left(N\right)$ cells. Again, the range is defined as the number of the highest occupied cell minus the lowest occupied cell. A non-directional test is obtained by simultaneously considering both cases and multiplying $\left(\alpha \right)$ by two to adjust for multiplicity. 

### 2.6. Computational Details 

*p*-Values for the weighted Fleming and Harrington LRT were computed using the “Test=FH” option in the strata statement of the LIFETEST procedure in SAS v.9.4 software (Cary, NC, USA), while *p*-values for the Max-Combo procedure were obtained iteratively [50]. The SAS code for performing the iLRT is provided in the Appendix A. In most cases, the computational run time for the iLRT is approximately 4-fold (or more) faster than the default 4-component Max-Combo test. 

*p*-Values ≤ 0.05 were deemed to be statistically significant. Unless otherwise indicated, computed values were presented to two significant digits using the Goldilocks (Efron–Whittemore) rounding method, rather than a fixed number of decimal places [51].

## 3. Examples

**Figure 1:**
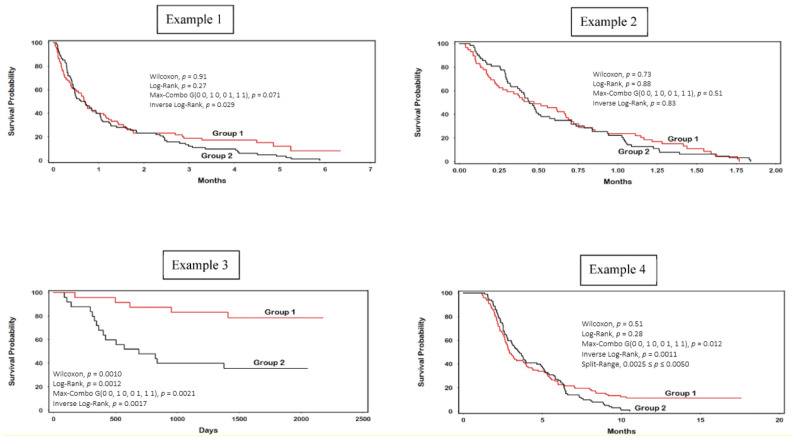
Kaplan–Meier curves corresponding to Examples 1–4.

Four examples are presented in this section comparing the results of the Prentice–Wilcoxon, standard Mantel (unweighted), combination Max-Combo (default four-component), and inverse log-rank tests. The combinatoric SRT is presented as a non-LRT comparison in the fourth example. Kaplan–Meier (product-limit) plots are provided for each example (see Figure 1). Summary computational results of the iLRT for the four examples are shown in Table 2.

### 3.1. Example 1

In this non-randomized cohort of *n* = 157 emulated patients with metastatic (stage IV), non-squamous cell lung cancer (NSCLC), who failed to respond to conventional chemotherapy, 75 opted to receive an experimental immune therapy compound (Group 1) versus 82 who were provided hospice care (Group 2) [46]. Among the 75 patients in the first group, 11 had censored outcomes, while all of the patients in Group 2 experienced an event (Table 2). Soon after the second month, a noticeable late survival advantage materialized for the experimental group, while those in the hospice group continued to decline (see Kaplan–Meier plot for Example 1). Notably, the Kaplan–Meier curves otherwise crisscrossed for the first two months before diverging. The median survival time for Group 1 was slightly higher than Group 2 (0.69 versus 0.65 months). Only the iLRT yielded a statistically significant survival group difference (*p* = 0.029). Although the default Max-Combo failed to achieve statistical significance (*p* = 0.071), several individual FH-LRT values for (ρ) and (γ) had correspondingly lower *p*-values than the iLRT, with a minimum being observed for (ρ = 0, γ = 5; *p* = 0.015) (Table 3). That is, the power of the Max-Combo test in a specific scenario may not exceed their component FH test statistics [52].

### 3.2. Example 2

The objective of Example 2 is to demonstrate the non-significant difference between the two treatment arms in Example 1, prior to their point of separation. As expected, upon deleting observations occurring after 1.9 months, none of the LRTs in this example had statistically significant *p*-values. The highest value *p*-value corresponded to the iLRT (*p* = 0.83), followed by the default Max-Combo test (*p* = 0.51). 

### 3.3. Example 3

An important characteristic of an omnibus LRT is the ability to accommodate late separating survival curves, while also having power to detect significant differences occurring from the beginning of a study. Example 3 elaborates on the comparative analysis of two cancer therapies, historically presented by Brown and Hollander [53]. Referring to the Kaplan–Meier plots for this example, we see that the treatment curves are relatively parallel, suggesting proportional hazards over time. Both the standard Mantel LRT (*p* = 0.0012) and Prentice–Wilcoxon LRT (*p* = 0.0010) are statistically significant, while the iLRT (*p* = 0.0017) and the default Max-Combo test (*p* = 0.0021) yield comparable levels of statistical significance, though to a slightly lesser degree. 

### 3.4. Example 4

Example 4 illustrates a special case of late separating survival curves, as originally presented by the author [49]. In this analysis, all the patients in the comparison arm (Group 2) experience the event of interest, while 11 of the patients in the experimental treatment arm (Group 1) have survival times greater than the last event in Group 2 at 9.5 years. Accordingly, the SRT is applicable in this example and yields a *p*-value of between 0.0025 and 0.0050, as there is one censored value at 3.0 years that occurs in Group 1 before the last event in Group 2. While all of the values in Group 1 beyond the completion of Group 2 are censored, an equivalent *p*-value would have been obtained for this degenerate case, even if one or more of these censored values were events (which is the case for LRTs in general). 

In this example, the *p*-value obtained for the SRT is comparatively close to the iLRT (*p* = 0.0011) and the default (four-component) Max-Combo procedure (*p* = 0.012), with the iLRT yielding the more statistically significant value. The cumulative frequency for the split-range test given *n* = 100 and *N* = 200 is provided in Table 4.

### 3.5. Comparison with the Cox Regression Model

In Examples 1, 2, and 4, which depict non-proportional hazards, the corresponding hazard ratios (HRs) and significance levels (estimated by a Cox regression model) were 1.2 (*p* = 0.27), 1.0 (*p* = 0.88), and 1.2 (*p* = 0.28), respectively. In contrast, the hazards for the two survival curves shown in Example 3 were relatively constant over time (HR = 0.22) and manifested a *p*-value of 0.0030, being slightly less significant but comparable to the iLRT (*p* = 0.0017) and default Max-Combo procedure (*p* = 0.0021). 

## 4. Sample Size and Power 

### 4.1. Sample Size and Power Methodology

To compute the sample size and power for a planned trial (i.e., how frequently a test will detect the falsehood of an underlying hypotheses when it is wrong), we note that [54]

(43) Ψ=Zα2+Zβ,

where $\left(\Psi \right)$ is the standardized test statistics for the iLRT, and $Z_{\alpha }$ denotes the 100(1 − α) percentile of a standard normal distribution and proceed in a manner comparable to Garès and colleagues [55]. Specifying the desired power as (1 −  β) for an α-level (two-sided) test of significance, the respective sample size for Group 1 $\left(N_{Total}=2\;\times \;N_{Group\;1}\right)$ is given as

(44)   N1=σZα2+Zβ∑i=1mlog⁡(Ri)Ridi1−Ri1diRi2,

where

(45) $$\sigma =\sqrt{R_{11}\sum_{i=1}^{m}{\left(\frac{log(R_{i})}{R_{i}}\right)}^{2}\sigma_{x_{i}}^{2}}.$$

Rearranging the formula for sample size, we see that

(46) Power=(1−β)=0.5+0.5erfN1σ∑i=1mlog⁡(Ri)Ridi1−Ri1diRi−Zα2/2,

where

(47) $$erf\left(z\right)=\frac{2}{\sqrt{\pi }}\int_{0}^{z}e^{{-t}^{2}}dt.$$

### 4.2. Sample Size and Power Example

In Example 1, the results of a non-randomized cohort were presented where a new experimental compound was compared with hospice care for late stage, refractory lung cancer. Based on the promising findings from this study, a pharmaceutical company would like to conduct a Phase-3 clinical trial randomizing an equal number of patients to the two treatment groups.

Specifically, the company wishes to reject the null hypothesis of equivalent survival times between the two arms of the planned study with a probability of 90% (given that the survival curves are truly different), and a Type I (two-sided) error rate of 5%. Plugging in the numbers from the first row of Table 1, we see that

(48) $$N_{1}={\left[\frac{\sqrt{\left(64+11\right)\;\left(0.33\right)}\left(1.96+1.3\right)}{\sqrt{1.6}}\right]}^{2}≅164.$$

Upon being informed of the sample size, management decided that the cost to conduct the trial would be too high. Instead, they suggested a trial of no more than 144 patients per arm and asked the statistician to determine the corresponding statistical power, computed as

(49) $$\mathrm{Power}=(1-\beta )=\left\{0.5+0.5erf\left[\left\{\left[\frac{\left(12\right)\left(1.26\right)}{4.97}\right]-1.96\right\}/1.41\right]\right\}\;≅73\%.$$

## 5. Discussion

### 5.1. Overview

The choice of weights for an LRT is arbitrary and largely predicated on the efficiency to detect treatment differences [56]. Under the null hypothesis, optimal “pre-specified” weights are a function of the total number of participants at risk at the time of a respective event and are estimated from the data [57]. While weighted rank tests are valid under unequal censoring, the asymptotic relative efficiency of the test statistic depends on the censoring distribution. A weighted LRT should be reasonably robust to unequal right-censoring, as permutation tests may fail to provide suitable approximations [58]. In such cases, the permutation computed variance may underestimate the true variance when censoring is unequal [59]. Additionally, the analysis of arbitrarily interval-censored survival data requires special techniques beyond that discussed here [60,61].

The optimal weight or combination of weights for an LRT has a defined power advantage, contingent upon advanced knowledge of when the survival curve separation will occur (e.g., early, mid, or late). Thus, the ideal selection depends on the data, knowledge of which may not be feasible before the completion of a study. While pilot data or results from comparable studies can be helpful in the decision-making process, there is no guarantee that a planned study will behave similarly. While several researchers have proposed adaptively choosing weights as a function of the data [38,47,62], the properties of such tests may be challenging to predict and may have less power when compared with the traditional unweighted LRT with proportional hazards [25]. 

The iLRT is nearly as powerful as the standard LRT under proportional hazards. Yet, the iLRT is more sensitive to time-dependent, non-proportional hazards observed for differential or single arm delayed treatment effects. When an investigator is uncertain in advance about the shape of the survival curves, it is not apposite to select an LRT after the data have been collected as the analytic method should be clearly specified in the protocol prior to the initiation of a study. One option is to select a combination of FH weights in the form of the Max-Combo test. While this procedure performs reasonably well, again as previously noted, it is possible to reject the null hypothesis both in favor and against a particular treatment for the same data [45]. Combination tests also may have diminished power, albeit marginal, to detect treatment differences, resulting from the implicit multiplicity correction required by the procedure. As a single weight method, the iLRT does not require adjustment for multiple testing and provides a flexible and non-subjective means for analyzing both continuing and late separating survival curves. However, if the investigator is certain of the shape of the survival curves in advance, then an appropriately parametrized FH-LRT may present the optimal choice for the planned analysis. 

### 5.2. Efficiency

The chi-square statistic $\left(\zeta_{w_{i}}\right)$,$\;\left[\hat{{w_{i}d}_{i}/R_{i}}\right]\;$is the minimum, best asymptotic normal (BAN) estimator for [$E_{i}\;$=$\;\left({w_{i}R}_{i1}d_{i}/R_{i}\right)$], providing that it is a consistent estimate of the latter and asymptotically normal under large sample conditions (with properties akin to the maximum likelihood estimator and Fisher’s information loss, albeit based on cell frequencies vs. original observations) [63,64,65]. Among all such asymptotically normal estimates within a multinomial framework, none have a smaller variance [66]. As such, $\left(\zeta_{w_{i}}\right)$ belongs to a class of tests which are unbiased and equivalent in limit to Neyman’s λ-test [54,67]. While tests within this family have comparable or more stringent power against Pitman alternatives (i.e., asymptotic relative efficiency), there is no guarantee that the statistic converges to a normal distribution at a reasonably fast rate, especially when observations are sparse toward the extreme right tail, with manifest censoring [68,69,70,71]. For Type II right-censored data with a presumed number of events, the total time of the trial is unknown until the last event occurs (versus trials with a fixed time of termination) [72]. Nonetheless, both types of censoring may lead to unreliable inferences and are challenging to model if censoring is sporadic, non-stationary, or a differential censoring mechanism exists between the two arms of a trial [73]. The misspecification of weights with respect to censoring or premature withdrawals can have undesirable and difficult to predict consequences on test efficiency and power, especially in the presence of incomplete data. 

### 5.3. Power

#### 5.3.1. Lakatos–Cantor Method for Computing Power

In practice, an alternative method for computing the power of weighted LRTs exists that only requires specifying the survival probabilities at designated times for the two arms being compared. This method (based on a seminal paper by Lakatos in 1988 and later simplified by Cantor for practical application) involves partitioning the study period into a set number of subintervals [74,75]. The survival distribution for each treatment group is approximated by a piecewise linear curve, with the respective hazard at each time point estimated by linear interpolation. A Markov chain process is used to model state transitions of events across time. When both the sample size and corresponding number of subintervals are reasonably large, the power obtained by this method will tend toward that described in Section 4 [76].

The advantage of the piecewise linear approach for determining power is that one can visually estimate the required survival probabilities from published Kaplan–Meier curves or, alternatively for smaller sample sizes, by the Nelson–Aalen method [77]. Furthermore, computer packages for implementing the Lakatos model, allowing for user-provided LRT weights, are readily available [78,79]. The main limitation of this method lies in partitioning the study period into subintervals (i.e., discretizing continuous data into bins), particularly when the number of subintervals is small. In this case, the resulting values within each subinterval can vary depending on how the boundaries for the subintervals are chosen and potentially bias the analysis (i.e., “Mendel effect”) [80,81]. Implementing a prescribed algorithm to choose the interval widths alleviates this concern to some degree. However, there is no consensus on the optimal vs. practical approach for binning, with some historic and hitherto commonly used procedures lacking statistical consistency [82,83,84,85,86]. 

#### 5.3.2. Interim Power and Sample Size Re-Estimation

While event level information often is not available during the planning stage of a clinical trial, investigators typically will have access to published Kaplan–Meier survival plots from previous studies [78]. A stop-gap measure, pending the availability of more precise information, involves initially estimating power using the Lakatos–Cantor method and then re-estimating the power and sample size at an interim point, implementing the iLRT method described in Section 4. Providing that the investigator and other members of the study team remain blinded, there is no need to apply a *p*-value penalty for each interim look at the data. 

A first interim analysis typically is conducted after more than half of the planned events in the trial have been observed, with less than ~6% (or a predetermined percentage) of participants being lost to follow-up or early censoring. In some cases, if allowed by the protocol and appropriately penalized, the unblinded “data monitoring committee statistician” may recommend a second sample size re-estimation after 75% of the planned events have occurred since sample sizes may have to be adjusted depending upon the point of late separation for the survival curves. Of note, “writing back” the time of censoring to the time of an earlier administrative event can lead to an artifactual late separation of survival curves or unintended differential bias [87].

### 5.4. Limitations 

#### 5.4.1. Potential Sources of Bias

Analogous to the broad class of tests for comparing survival time differences between the two arms of a study, results of the iLRT may yield biased results if censoring is related to prognosis or if survival probabilities are not stationary and instead depend upon when a participant is recruited into the clinical trial [88]. Likewise, the iLRT may experience a significant loss of power if competing risks are not independent or censoring is informative (i.e., a correlation exists between censoring and the event of interest) [89]. Examples include drug withdrawal attributable to a lack of efficacy or intolerability. Furthermore, as a test of statistical significance, the iLRT is not designed to estimate the effect size for a treatment difference between groups or to compute confidence intervals of an effect [88].

While the objective of the iLRT is to reduce the false negative rate while achieving a statistically significant result, the procedure may experience a slight loss of power in the case of diminishing treatment effects, where the survival curves initially diverge but converge back together over time. If this is anticipated and the clinician has a specific interest in diminishing treatment effects, then the Max-Combo or FH (1,0) tests may represent a better choice for accommodating this possibility. When the curves extend beyond the point of diminishing treatment effect and then crossover, this poses interpretational challenges that may be best handled as a post hoc stratified analyses. The latter scenario merits exploring the underlying reasons for the crossing-over and any subgroup effects (e.g., potential treatment switching) before reaching any conclusions [7,90]. In the case of crossing hazards, a two-sample semiparametric procedure has been proposed as an alternative analytic approach [91]. Investigators also may consider the use a “standard of care reference arm” with a comparable hazard pattern. 

A weighted LRT that is not consistent under stochastic ordering may not necessarily control the Type I error rate [92]. In Example 4, with the SRT as a comparison technique, we provide a heuristic argument that both the iLRT and default Max-Combo test independently control Type I error to within an absolute difference less than or equal to 0.0039 in the case of late separating survival curves, while preserving the false positive rate under proportional hazards (Example 3). Analogous to the consistent Prentice–Wilcoxon statistic, the weight for the iLRT is based upon the number of participants at risk for each time point. By taking the logarithm of the number at risk and scaling accordingly, the iLRT is bounded above by the Prentice–Wilcoxon test. 

When censoring is not under the control of the investigator, censored participants may not have the same future risk of the outcome event as non-censored participants [93]. Consequently, there may not be a one-to-one correspondence between cause-specific hazard and cumulative incidence [94]. Such non-informative censoring can occur under competing risks and potentially bias risk estimates [95]. Unfortunately, commonly used methods to account for non-competing risks depend on the hazards being proportional, which may not always be the case when using the iLRT or other weighted procedures [96]. When appropriate, competing risks can be treated as random effects in a multilevel, mixed-effects model. 

#### 5.4.2. Sparseness of Data and Small Sample Sizes

The iLRT may lack statistical power if few events accompany the divergence of treatment hazards or censoring is heavy [97]. Sparseness in the tails of the survival curves at the time of interim analysis also can hinder reliable sample size re-estimation. As asymptotic theory was used to establish limiting formulas, the small-sample behavior of the iLRT may be uncertain in such cases. With sparse data, bootstrapping or permutation methods may be considered for validating the model robustness of the iLRT.

#### 5.4.3. Computational Barriers

Standard available commercial software to compute power for weighted LRTs using the Lakatos–Cantor method generally are limited to a few weight options (e.g., standard log-rank, generalized Wilcoxon/Gehan–Breslow, and Tarone–Ware). However, a downloadable computer algorithm to compute the Lakatos–Cantor method for the iLRT and other user specified weights is available online [75].

### 5.5. Future Directions

The basis of this manuscript relies on selected empirical examples to support the use of the iLRT. Other situations may necessitate a different approach, and future research will help to delineate the most appropriate solution, such as adaptive or machine learning strategies [8,38,47]. The restricted mean survival times (RMSTs) method, which visually corresponds to the area under the Kaplan–Meier curve for a specified time period (τ), is another method for analyzing non-constant hazards and may be useful as secondary analysis [45,98]. However, this technique depends on the arbitrary choice of (τ). The misspecification of this value can yield statistically significant but clinically irrelevant results by focusing only on a particular region of the survival curves. Exploring an assortment of data-driven (τ) points and accounting for these choices when estimating statistical significance is a promising area of ongoing research. Piecewise proportional hazard models also may be a good choice in some cases [99,100], and the hyperbolic cosine and logistic-like weight functions have received mention in the literature [52]. 

While a diverse array of weights and variance estimators have been proposed for the LRT, there is a paucity of comparative information regarding their versatility and efficiency under varying levels of non-proportionality, censoring, and competing risks [36,55,59,101]. Furthermore, when the event rate is low, weighted LRTs may not retain their range of flexibility [102]. Future analysis, beyond the scope of the current manuscript, may be merited. 

## 6. Conclusions

A truly omnibus test is able to accurately detect survival differences over the clinical spectrum of a drug trial, regardless of whether a positive result is apparent from the start of therapy or only materializes later in the study (i.e., there is a time lag in the effectiveness of therapy). In contrast to the standard LRT, which treats all time points uniformly, an appropriately weighted LRT has the advantage of identifying significant delayed treatment effects with only a slight reduction in power for other survival outcomes. That is, under proportional hazards, with a nominal decrease in the probability of truly rejecting the null hypothesis, a substantial gain in efficiency for late separating survival curves is achieved [103].

While the quest for a “Holy Grail” test with infinite flexibility (i.e., immune to the type of non-proportional hazard) remains elusive, the single-weight iLRT possesses many of the desirable properties of such an omnibus method, particularly when the terminal event of one arm occurs before study completion. The iLRT equals or surpasses the default (four-component) Max-Combo method in many important applications and is objectively simple to implement with available computer code. The method does not require complex or timely simulations to estimate study power, and as a single-weight test, the iLRT does not involve implicit multiplicity correction nor depends on the arbitrary selection of weights. Nonetheless, in some cases, the iLRT may lack the flexibility and power of other more generalized multi-component Max-Combo tests or individual (two-parameter) Fleming–Harrington (FH) weights.

Relying entirely on a proportional hazards assumption when planning for and selecting a statistical test is unwise unless one is highly confident about the parallel shape of the ensuing hazard functions [98]. For example, the benefit of treatment may not occur immediately, but rather require a certain amount of time to overcome a lengthy disease period. A delayed treatment benefit also may be a consequence of “immunologic adjustment”, which often occurs with certain newer-generation cancer drugs. In contrast, the antibiotic treatment of an infectious disease generally manifests a rapid treatment response. 

The single-weight iLRT does not depend on an arbitrary choice of weights yet is relatively versatile and retains excellent power under delayed treatment effects. Nonetheless, a preponderance of investigators continue to use the more familiar assumption of constant event rates and proportional hazards in the design and analysis of randomized controlled trials, despite a potential loss of power and efficiency if this supposition does not hold [104].

## Figures and Tables

**Table 1:** Events and non-events in the risk set at ($t_{i}$) by study group.

	Group 1	Group 2	Total
Event	$d_{i1}$	$d_{i2}=d_{i}-d_{i1}$	$d_{i}$
Non-event	$R_{i1}-d_{i1}$	$R_{i2}-d_{i2}$	$R_{i}$ − *d_i_*
Total	$R_{i1}$	$R_{i2}=R_{i}-R_{i1}$	$R_{i}$

**Table 2:** Summary computations for the inverse log-rank test (Examples 1–4).

Ex.	Group 1		Group 2		Numerator ${\left[\sum_{i=1}^{m}w_{i}\left(d_{i1}-\mu_{x_{i}}\right)\right]}^{2}$	Denominator $\sum_{i=1}^{m}{\left(w_{i}\right)}^{2}\sigma_{x_{i}}^{2}$	$\chi_{1}^{2}$	*p* *
	Event	Censored	Event	Censored				
1	64	11	82	0	1.6	0.33	4.8	0.029
2	57	2	63	0	0.017	0.37	0.046	0.37
3	16	9	5	19	0.46	0.047	9.9	0.0017
4	88	12	100	0	2.0	0.19	11	0.0011

* *p*-Values computed using non-rounded values. Ex. = Example. m= # of time points; $w_{i}=\frac{\log\left(R_{i}\right)}{R_{i}}$; $\mu_{x_{i}}=\frac{{R_{i1}d}_{i1}}{R_{i}}$; ${\sigma_{x_{i}}^{2}=R}_{i1}\left[\frac{d_{i}}{R_{i}}\right]\left[1-\left(\frac{d_{i}}{R_{i}}\right)\right]\left[\frac{R_{i}-R_{i1}}{R_{i}-1}\right]$; $\chi_{1}^{2}$ = Numerator/Denominator.

**Table 3:** Individual FH-LRT *p*-values for G(ρ,γ).

G(0, γ)	*p*-Value	G(ρ,5)	*p*-Value
(0,0)	0.27	(0,5)	0.015
(0,1)	0.033	(1,5)	0.44
(0,5)	0.015	(5,5)	0.35
(0,10)	0.021	(10,5)	0.18
(0,15)	0.036	(15,5)	0.47
(0,20)	0.053	(20,5)	0.85
(0,25)	0.069	(25,5)	0.42

**Table 4:** Cumulative frequency for the split-range test (n = 100, N = 200).

r	$P\left(R=r\right)$	$P\left(R\leq r\right)$	r	$P\left(R=r\right)$	$P\left(R\leq r\right)$
184	0.00008	0.00016	192	0.01498	0.03243
185	0.00016	0.00032	193	0.02677	0.05920
186	0.00032	0.00064	194	0.04662	0.10582
187	0.00063	0.00127	195	0.07851	0.18434
188	0.00122	0.00249	196	0.12627	0.31060
189	0.00234	0.00483	197	0.18940	0.50000
190	0.00441	0.00924	198	0.25126	0.75126
191	0.00821	0.01745	199	0.24874	1.0000

## Data Availability

Data for Example 1 is provided in Appendix B. Please refer to indicated references for data sources of the other examples.
